# Word-Level Motion Learning for Contactless QWERTY Typing with a Single Camera

**DOI:** 10.3390/s26041087

**Published:** 2026-02-07

**Authors:** Sung-Sic Yoo, Heung-Shik Lee

**Affiliations:** Department of Smart Mobility Engineering, Joongbu University, 305 Dongheon-ro, Deogyang-gu, Goyang-si 21713, Gyeonggi-do, Republic of Korea; sungsicyoo@gmail.com

**Keywords:** contactless text entry, word-level typing, QWERTY keyboard, hand motion learning, monocular RGB camera, vision-based typing

## Abstract

Contactless text entry is increasingly important in immersive and constrained computing environments, yet most vision-based approaches rely on character-level recognition or key localization, which are fragile under monocular sensing. This study investigates the feasibility of recognizing natural QWERTY typing motions directly at the word level using only a single RGB camera, under a fixed single-user and single-camera configuration. We propose a word-level contactless typing framework that models each word as a distinctive spatiotemporal finger motion pattern derived from hand joint trajectories. Typing motions are temporally segmented, and direction-aware finger displacements are accumulated to construct compact motion representations that are relatively insensitive to absolute hand position and typing duration within the evaluated setup. Each word is represented by multiple motion prototypes that are incrementally updated through online learning with a trial-delayed adaptation protocol. Experiments with vocabularies of up to 200 words show that the proposed approach progressively learns and recalls word-level motion patterns through repeated interaction, achieving stable recognition performance within the tested configuration at realistic typing speeds. Additional evaluations demonstrate that learned motion representations can transfer from physical keyboards to flat-surface typing within the same experimental setting, even when tactile feedback and visual layout cues are reduced. These results support the feasibility of reframing contactless typing as a word-level motion recall problem, and suggest its potential role as a complementary component to character-centric camera-based input methods under constrained monocular sensing.

## 1. Introduction

Text entry is one of the most fundamental and frequently performed tasks in human–computer interaction. With the rapid expansion of computing platforms from desktop environments to mobile devices, wearables, and immersive systems such as virtual and augmented reality (VR/AR) [1,2], the demand for flexible and unobtrusive text input techniques has increased substantially. In such contexts, conventional physical keyboards impose spatial constraints, disrupt immersion, and require dedicated surfaces, making them poorly suited for emerging interaction scenarios. As a result, contactless text entry methods that operate without physical contact or specialized hardware have become an important and active research topic [3,4,5].

Among contactless approaches, virtual keyboard–based mid-air typing has been the most extensively studied [6,7,8]. These methods typically assume an imaginary QWERTY keyboard in mid-air and attempt to recognize individual characters by estimating fingertip positions or key regions. Owing to their conceptual similarity to conventional typing, such approaches offer a relatively low learning barrier and immediate familiarity for users. However, they fundamentally rely on accurate key-level localization and character-level decoding. Even small uncertainties caused by hand-tracking noise, viewpoint variation, or finger occlusion can lead to character-level errors, which then accumulate at the word level and significantly degrade overall text entry performance [5,9].

The limitations of character-centric recognition become particularly pronounced when contactless typing is implemented using a single RGB camera. On a physical QWERTY keyboard, adjacent keys are separated by only a few millimeters, while natural typing involves fast, simultaneous movements of multiple fingers with frequent inter-finger occlusion. Under these conditions, reliably discriminating fine-grained finger position differences for individual key inference requires extremely high spatial precision and careful calibration [10,11]. Such requirements are difficult to satisfy with monocular vision alone due to depth ambiguity and perspective distortion [12,13]. Consequently, key-centric or character-centric strategies are inherently fragile in vision-based contactless typing systems, particularly due to their strong reliance on precise sensing conditions and calibration, and their susceptibility to error accumulation from low-level localization failures.

To mitigate these challenges, another line of research has explored gesture-based and word-gesture typing approaches, in which continuous hand motions are interpreted as holistic gestures rather than sequences of discrete characters. By modeling entire words as motion trajectories, these methods can partially alleviate the error accumulation inherent in character-level decoding [14,15]. However, many existing gesture-typing systems rely on additional sensing hardware, assume specific device configurations, or require gesture styles that deviate substantially from natural QWERTY typing behavior, thereby limiting their general applicability, especially in terms of increased user burden, non-natural interaction styles, or dependence on additional sensing hardware and specific device configurations.

Meanwhile, advances in real-time hand pose estimation from monocular RGB input have reached a level of maturity that enables stable tracking of hand and finger joints without specialized sensors [16,17,18]. Leveraging this capability, recent studies have investigated vision-based text entry by recognizing typing on physical surfaces using RGB video or by inferring touch events and text input from egocentric camera views. Despite these advances, most existing vision-based approaches still focus on key-level decisions or character recognition, such as keystroke detection or air-writing, and relatively few treat the word itself as the primary recognition unit.

Behavioral biometrics research has demonstrated that typing patterns contain distinctive motion characteristics that can identify individual users [19,20,21]. Keystroke dynamics, which captures the temporal and spatial patterns of typing behavior, has been successfully applied to user authentication and identification tasks [22,23]. These findings suggest that finger motion patterns during typing are sufficiently discriminative to enable word-level recognition, yet this potential has remained largely unexplored in contactless text entry systems.

Taken together, existing contactless text entry research can be broadly categorized into (i) virtual keyboard approaches based on key-level or character-level recognition and (ii) gesture-based approaches that often rely on additional sensors or specialized interaction techniques. However, direct word-level recognition using only a single RGB camera—while preserving natural QWERTY typing motions and completely avoiding character decoding or key localization—remains largely underexplored. From a methodological standpoint, many prior contactless text entry systems rely on character-level classification pipelines or sequence-matching techniques such as DTW or HMMs, often combined with explicit language models to correct low-level recognition errors. In contrast, the proposed framework intentionally avoids character decoding, temporal alignment, and language-model support, focusing instead on direct word-level motion recall under minimal sensing assumptions. As a result, direct baseline comparisons with character-centric or sequence-alignment-based methods are not straightforward and are outside the scope of this feasibility-oriented study.

To address this gap, we reformulate contactless typing not as a character recognition problem, but as a word-level motion recall problem. The proposed system models each word as a distinctive spatiotemporal finger motion pattern derived from hand joint trajectories captured by a single RGB camera. Continuous hand movements are temporally segmented, and direction-aware finger displacements are accumulated to construct compact motion representations that are relatively insensitive to absolute hand position and typing speed within the evaluated single-user, fixed-camera configuration, while remaining discriminative across words.

Furthermore, recognizing that natural typing exhibits substantial intra-word variability across repetitions [24], each word is represented by a set of motion prototypes rather than a single template. These prototypes are updated online: new prototypes are created only when novel execution patterns are observed, while existing ones are gradually adapted to reflect long-term user behavior. To prevent optimistic bias during evaluation, we introduce a trial-delayed learning protocol that explicitly separates prediction from adaptation within each trial.

In summary, this work proposes a contactless text entry framework that uses only a single RGB camera to directly recognize word-level motion patterns while preserving natural QWERTY typing behavior. By redefining the input unit from characters to words, the proposed approach opens a feasibility-oriented design space for vision-based text entry systems, in which robustness and scalability can be explored under constrained monocular sensing configurations.

Throughout this paper, claims of insensitivity or robustness are intended to be interpreted strictly within the constrained single-user, fixed-camera experimental regime evaluated, rather than as intrinsic properties of the representation.

The main contributions of this paper are threefold:

(1) We introduce a word-level contactless typing paradigm that operates using a single RGB camera without character decoding or key localization.
(2) We propose a temporally segmented, direction-aware motion representation combined with an online multi-prototype learning strategy to handle execution variability and user adaptation.
(3) We validate the feasibility of the proposed approach and explore scalability trends through systematic experiments with increasing vocabulary sizes.

## 2. Materials and Methods

This section describes the proposed word-level contactless QWERTY typing framework in detail. The core idea of the proposed method is to model an entire typing action as a word-level hand motion pattern, rather than decoding individual characters or estimating key locations. The framework operates with a single RGB camera and incrementally adapts to user-specific typing behaviors through online learning.

### 2.1. System Overview

**Figure 1:**
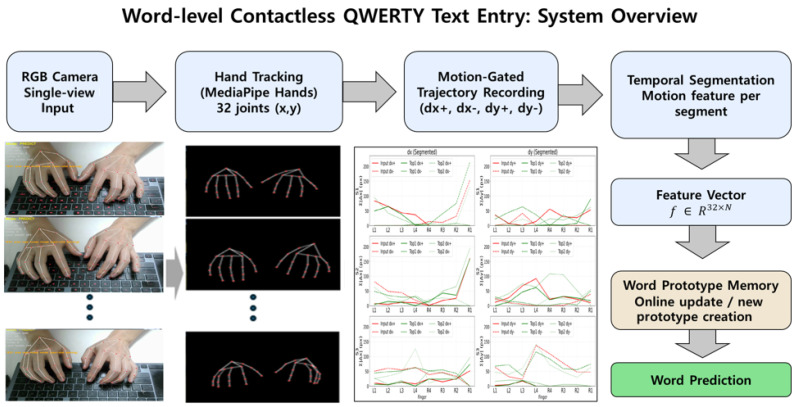
Overview of the proposed word-level contactless typing framework. The system processes monocular RGB input through hand tracking, motion segmentation, feature construction, word-level classification, and online adaptation.

The proposed system is a unified framework that performs both training and inference directly from monocular RGB input. As illustrated in Figure 1, the processing pipeline consists of six main stages: (i) real-time hand landmark tracking, (ii) motion-gated extraction of word-level input segments, (iii) temporal segmentation of motion sequences, (iv) segment-wise motion feature construction, (v) word-level classification via multi-prototype matching, and (vi) online prototype adaptation.

Unlike conventional text-entry approaches that rely on character-level recognition or explicit key localization, the proposed framework directly associates each word with a distinctive spatiotemporal hand motion pattern. This design enables users to perform natural QWERTY typing motions while allowing the system to learn and refine word representations through repeated executions.

**Figure 2:**
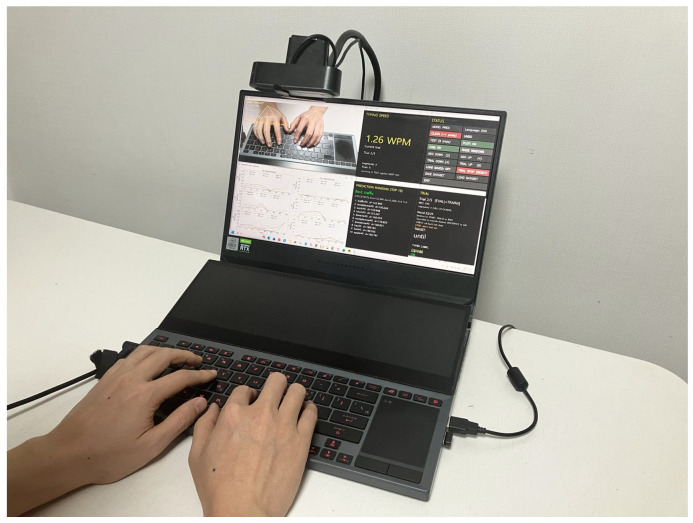
Experimental setup for the proposed contactless typing system. A single RGB camera is mounted above the keyboard to capture hand and finger motions during natural QWERTY typing. The system performs real-time hand landmark tracking, motion segmentation, and word-level prediction within a unified interface.

The physical experimental setup corresponding to this pipeline is shown in Figure 2. A single RGB camera is mounted above the keyboard to capture hand and finger motions during natural typing. The camera remains fixed throughout all experiments, ensuring a consistent viewpoint and scale. The captured video stream is processed in real time to extract hand landmarks, segment typing motions, and perform word-level prediction and adaptation within a unified graphical interface. This setup reflects the minimal sensing assumptions of the proposed framework and serves as the basis for all experiments reported in this paper.

### 2.2. Hand Landmark Representation

Hand landmarks are extracted in real time using MediaPipe Hands [18]. For each frame t, we track four fingers (index, middle, ring, and pinky) on both hands, and select four joints per finger (MCP, PIP, DIP, TIP). This yields a total of $N_{p}=32\;(8\;\mathrm{fingers}\times 4\;\mathrm{joints}),$

2D landmark points represented in image coordinates:

(1) $$p_{i}(t)=(x_{i}(t),y_{i}(t)),i=1,\ldots ,N_{p},$$

The thumb is excluded because it primarily contributes to non-alphanumeric actions (e.g., space/shortcut behaviors) and its motion tends to introduce variability that is not essential for distinguishing word-level QWERTY motion patterns in our setting. Using multiple joints per finger, rather than fingertip-only tracking, improves robustness to articulation differences and allows the representation to reflect both translational and articulatory finger motions.

**Figure 3:**
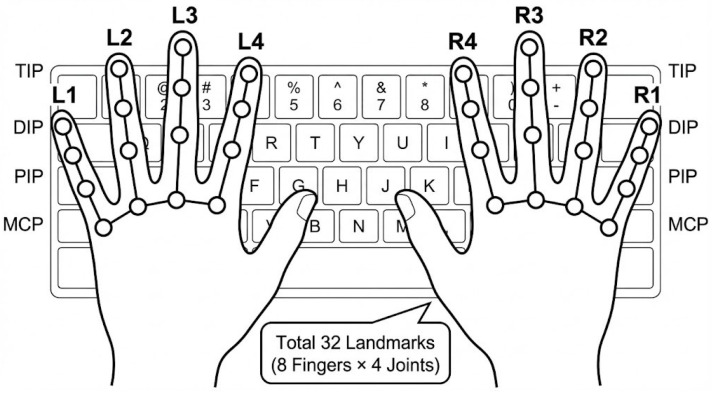
Hand landmark configuration used for word-level motion representation. Four fingers (index, middle, ring, and pinky) are tracked on both hands, with four joints per finger (MCP, PIP, DIP, and TIP), resulting in a total of 32 landmarks. The thumb is excluded. Landmark indices follow a fixed finger-wise ordering consistent with the QWERTY layout.

In addition, the landmark ordering follows a fixed finger-wise layout consistent with the QWERTY structure used throughout this study: [L1, L2, L3, L4, R4, R3, R2, R1] where L1–L4 denote the left-hand index-to-pinky fingers and R1–R4 denote the right-hand index-to-pinky fingers (reported in reverse to maintain a symmetric left-to-right arrangement in subsequent feature plots; see Figure 2).

(2) $${\tilde{p}}_{i}(t)=\alpha {\tilde{p}}_{i}(t-1)+(1-\alpha )p_{i}(t),$$

where $\left.\alpha \in [0,1\right)\;$ is the temporal smoothing factor. We apply an exponential moving average to suppress high-frequency landmark noise, yielding smoothed landmarks ${\tilde{p}}_{i}(t)$. The selected landmark configuration and joint indexing are illustrated in Figure 3.

### 2.3. Motion-Gated Word Recording

#### 2.3.1. Motion-Based Start Detection

To distinguish intentional typing motions from idle hand poses, word-level motion recording is initiated using a motion gate based on inter-frame landmark displacement. At frame t, the maximum displacement across all tracked landmarks is defined as

(3) $$d(t)=\underset{i}{max}\;{∥p_{i}(t)-p_{i}(t-1)∥}_{2},$$

where $p_{i}(t)$ denotes the 2D position of landmark i  at time t.

Recording begins when d(t) exceeds a predefined threshold $\tau_{\mathrm{start}}$, indicating the onset of deliberate finger motion. This motion-gated mechanism automatically excludes static hand poses and transitional periods preceding typing, thereby improving the temporal consistency and stability of the extracted motion features.

#### 2.3.2. Event-Driven Termination

The termination of word-level motion recording is controlled by explicit user-driven events, and the strategy differs depending on whether a physical keyboard is available. This design allows the system to maintain precise experimental control during training while supporting fully contactless operation during prediction.

During training and controlled test sessions conducted on a physical QWERTY keyboard, word boundaries are explicitly defined using the space key. This keyboard-based termination provides clear and reliable segmentation of word-level input, ensuring accurate supervision for learning word-specific motion prototypes and preventing ambiguity in word boundary definition during evaluation.

In contrast, during keyboard-free prediction scenarios on flat surfaces, word termination is detected using an acoustic trigger. A built-in microphone continuously monitors ambient sound intensity, and a word is finalized when a short impulse sound—typically produced by a deliberate thumb tap on the surface—exceeds a predefined amplitude threshold. To improve robustness against background noise and unintended activations, hysteresis and cooldown mechanisms are applied to the acoustic trigger.

The acoustic signal was sampled at the system audio sampling rate, and word termination was triggered when the short-time audio amplitude exceeded a predefined threshold. To suppress spurious activations caused by background noise, a simple hysteresis mechanism and a fixed cooldown interval were applied between successive triggers. No advanced noise modeling was employed, as the acoustic trigger was used solely to provide a reliable word boundary during feasibility evaluation. By combining motion-gated onset detection with event-driven termination, the proposed system can provide controlled temporal segmentation of continuous hand motion into discrete word-level input units under the evaluated experimental conditions. The termination mechanisms were introduced to ensure reliable and unambiguous word boundary definition during feasibility evaluation and were not intended to represent practical or fully contactless segmentation solutions. Quantitative analysis of segmentation errors, false triggers, and termination robustness is outside the scope of the present study and is left for future work.

### 2.4. Temporal Segmentation and Feature Construction

#### 2.4.1. Uniform Temporal Segmentation

Given a trajectory consisting of T  frames, the time axis is uniformly partitioned into N segments, where N is a user-defined positive integer. Segment m spans the frame index range

(4) $$\left[\left\lfloor \frac{mT}{N}\right\rfloor ,\;\left\lfloor \frac{\left(m+1\right)T}{N}\right\rfloor \right],\;\;\;\;m=0,1,\ldots ,N-1.$$

This uniform segmentation preserves coarse temporal ordering of the typing motion while reducing sensitivity to absolute typing duration within the tested speed range. The value of N controls the temporal resolution of the representation and can be adjusted depending on the desired trade-off between feature granularity and dimensionality.

#### 2.4.2. Frame-to-Frame Displacement Computation

For feature construction, only three distal joints (PIP, DIP, and TIP) are used for each finger, while the MCP joint is excluded. Direction-aware accumulations of Δx and $\Delta y\;\left(x^{+},x^{-},y^{+},y^{-}\right)\;$ are computed by summing signed displacements across the selected joints. The MCP joint is excluded to reduce sensitivity to global hand translation and palm-level motion, which are less discriminative for word-level QWERTY typing and tend to introduce additional variability.

Let

(5) $$\Delta x_{i}(k)=x_{i}(k+1)-x_{i}(k),\Delta y_{i}(k)=y_{i}(k+1)-y_{i}(k)$$

denote the horizontal and vertical displacement of joint i between consecutive frames at time index k. To suppress micro-jitter caused by landmark noise and intermittent tracking fluctuations, displacement vectors whose squared magnitude satisfies

(6) $$\Delta x_{i}(k{)}^{2}+\Delta y_{i}(k{)}^{2}<\tau_{\mathrm{jit}}^{2}$$

are ignored.

#### 2.4.3. Direction-Aware Displacement Accumulation

For each finger f and temporal segment m, accumulated motion is computed using a direction-aware formulation that separates positive and negative displacement components along each axis:

(7) $$F_{f,x^{+}}^{\left(m\right)}=\sum_{i\in N_{f}}\sum_{k\in m}max(\Delta x_{i}(k),0),$$

(8) $$F_{f,x^{-}}^{\left(m\right)}=\sum_{i\in N_{f}}\sum_{k\in m}max(-\Delta x_{i}(k),0),$$

(9) $$F_{f,y^{+}}^{\left(m\right)}=\sum_{i\in N_{f}}\sum_{k\in m}max(\Delta y_{i}(k),0),$$

(10) $$F_{f,y^{-}}^{\left(m\right)}=\sum_{i\in N_{f}}\sum_{k\in m}max(-\Delta y_{i}(k),0),$$

where $N_{f}$ denotes the set of selected joints for finger f.

This formulation extends conventional unsigned displacement accumulation by preserving directional asymmetry in finger trajectories.

#### 2.4.4. Segment-Level and Word-Level Feature Construction

Each finger contributes four directional components $\left(x^{+},x^{-},y^{+},y^{-}\right)$ per segment. Concatenating all eight fingers yields a 32-dimensional segment-level feature vector:

(11) $$f^{\left(m\right)}\in R^{32}.$$

The final word-level motion representation is obtained by concatenating segment-level features across all temporal segments:

(12) $$x=[f^{\left(0\right)};f^{\left(1\right)};\ldots ;f^{\left(N-1\right)}]\in R^{32N}.$$

This representation captures finger-specific motion magnitude and directional structure while remaining relatively insensitive to absolute hand position and motion duration under the fixed camera geometry, frame rate, and typing surface used in this study.

**Figure 4:**
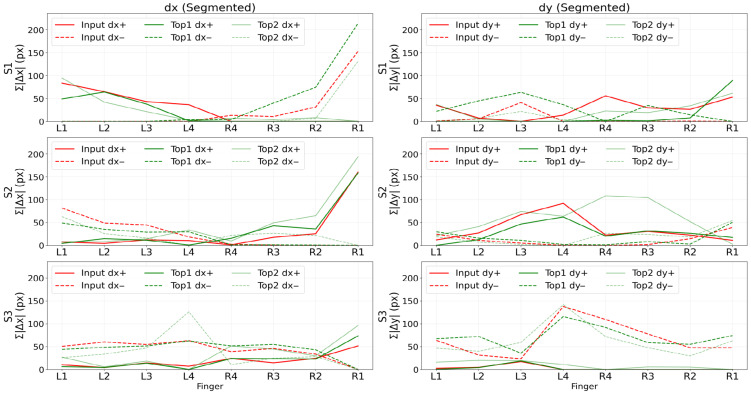
Illustrative example of word-level motion comparison for the word *encouragement*. Red solid and dashed curves denote the accumulated positive and negative dx (or dy) components of the input motion across fingers (L1–R1) and temporal segments (S1–S3). Green solid and dashed curves show the corresponding motion profiles of the closest predicted word prototype, highlighting the similarity of finger-wise directional motion patterns used for word matching.

To illustrate how this representation characterizes word-specific motion patterns, Figure 4 presents an example in which the word encouragement is typed and subsequently predicted based on hand motion alone. In the figure, the red solid and dashed curves represent the accumulated positive and negative components of dx (or dy) for each finger within each temporal segment of the input motion. The green solid and dashed curves depict the corresponding motion profiles of the closest predicted word prototype.

The strong structural similarity observed across segments and fingers indicates that each word is associated with a distinctive and repeatable hand motion pattern. By comparing directional motion distributions at the finger and segment levels, the proposed representation effectively assigns discriminative motion signatures to individual words, enabling robust matching between incoming hand motions and learned word-level prototypes.

### 2.5. Word-Level Classification via Multi-Prototype Matching

Each word class w is represented by a set of motion prototypes that model natural variability in word-level typing execution. Let

(13) $$P_{w}=\{p_{w,1},p_{w,2},\ldots ,p_{w,K_{w}}\}$$

denote the prototype set associated with word w, where each prototype $p_{w,j}$ corresponds to a previously observed word-level motion feature vector constructed as described in Section 2.4.

Given an input feature vector x, the distance between x and word w is defined as the minimum Euclidean distance to its prototypes:

(14) $$d\left(w;x\right)=\underset{j}{\min\;}{∥x-p_{w,j}∥}_{2}.$$

The predicted word is then obtained by

(15) $$\hat{w}=arg\;\underset{w}{min}\;d(w;x).$$

Allowing multiple prototypes per word enables the system to capture intra-word variability arising from differences in typing speed, finger articulation, and execution style across repetitions. Rather than enforcing a single canonical motion pattern, the proposed formulation models each word as a compact set of representative motion instances, resulting in more robust word-level classification under natural typing conditions.

### 2.6. Online Multi-Prototype Adaptation

To accommodate intra-word execution variability and gradual changes in user behavior over time, the proposed framework employs an online multi-prototype adaptation strategy that allows multiple prototypes per word during feasibility evaluation, without imposing an explicit upper bound in the current implementation. This design enables the system to incrementally model diverse execution styles without enforcing a single canonical motion pattern.

Given a training sample x associated with word w, the nearest prototype $p_{w,j^{*}}$ is first identified according to the distance measure defined in Section 2.5. If the distance between x and $p_{w,j^{*}}$ exceeds a predefined threshold $\tau_{\mathrm{new}}$, a new prototype is created and appended to the prototype set of word w. Otherwise, the selected prototype is updated using an exponential moving average:

(16) $$p_{w,j^{*}}\leftarrow p_{w,j^{*}}+\eta (x-p_{w,j^{*}}),$$

where $\left.\eta \in (0,1\right]\;$ denotes the update gain controlling the adaptation rate.

This incremental update mechanism enables continuous personalization to user-specific typing behavior while avoiding the need for batch retraining. By selectively creating new prototypes only when necessary, the system balances adaptability and stability, allowing previously learned motion patterns to be preserved. The absence of an explicit bound on the number of prototypes reflects a deliberate design choice aimed at evaluating feasibility under limited vocabulary and single-user conditions. In realistic long-term use, unbounded prototype growth would need to be addressed through standard mechanisms such as prototype pruning, consolidation, or memory-aware adaptation, which are left for future work.

### 2.7. Trial-Based Evaluation with Delayed Learning

System performance is evaluated using a trial-based repeated word-entry protocol. Each evaluation session consists of multiple trials in which the same target words are repeatedly entered by the user. The first trial is used solely for model initialization, during which motion samples are collected to establish an initial set of prototypes without performing recognition.

From the second trial onward, the system operates in a combined prediction-and-learning mode. To prevent evaluation bias caused by within-trial adaptation, a trial-delayed learning strategy is employed. Specifically, at the beginning of trial t, a frozen snapshot of the prototype set learned up to trial t − 1 is used exclusively for prediction. During trial t, newly observed samples are applied only to the live model and do not affect predictions within the same trial. As a result, learning effects from trial t influence recognition performance starting from trial t + 1.

**Figure 5:**
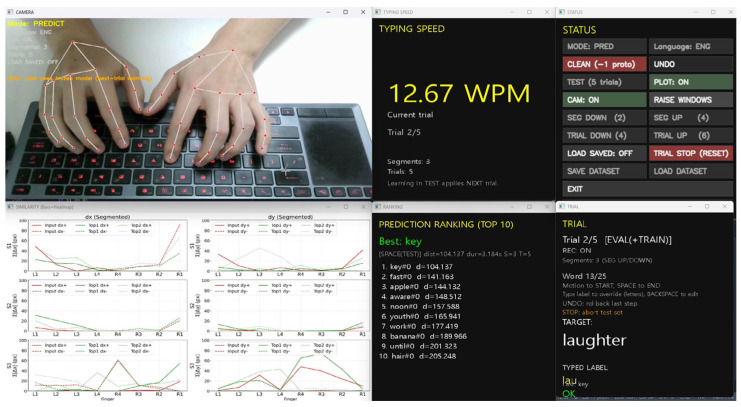
Integrated system interface used for trial-based evaluation with delayed learning. The interface visualizes real-time hand landmark tracking, segment-wise motion similarity to stored word prototypes, and prediction results. Word ranking, typing speed (WPM), and trial-level feedback are updated after each word entry, while model updates are applied with a one-trial delay to ensure unbiased evaluation.

This protocol ensures that recognition accuracy measured within each trial reflects genuine generalization to previously unseen executions, rather than immediate memorization effects. The overall evaluation workflow—including real-time motion capture, prediction feedback, and delayed model updates—is illustrated in Figure 5.

Recognition performance is reported as word-level accuracy, defined as the ratio of correctly predicted words to the total number of word-entry attempts. Typing efficiency is quantified using words per minute (WPM), computed based on the elapsed time between motion onset and termination for each word entry.

Figure 5 shows the integrated system interface used during trial-based evaluation. The upper-left panel visualizes real-time hand landmark tracking over a physical QWERTY keyboard captured by a single RGB camera. The lower-left panel presents segment-wise motion similarity curves that compare the current input motion with stored word prototypes, illustrating how directional motion features evolve across temporal segments. The right-hand panels display system status and evaluation feedback, including the current trial index, recording mode, segmentation configuration, and recognition results. The predicted word ranking is updated in real time, with the best-matching candidate highlighted, while typing speed (WPM) and trial-level recognition outcomes are reported after each word entry.

### 2.8. Implementation Details and Parameter Settings

All experiments were conducted under a fixed camera configuration. A single RGB camera with a resolution of 2560 × 1944 pixels (QHD, 1944 p) was mounted above the typing surface at a fixed height and orientation. The frame rate was fixed at 30 fps throughout all experiments.

System parameters were determined empirically through pilot experiments and were not tuned on a per-user basis, to evaluate the robustness of the proposed method under a common parameter configuration.

Table 1 summarizes the parameters used in the proposed system. Unless otherwise stated, all parameters were fixed throughout all experimental trials.

The number of temporal segments N  controls the temporal resolution of the motion representation and can be adjusted depending on the desired trade-off between discriminative power and feature dimensionality.

All remaining parameters were fixed throughout the study to isolate the effects of motion representation and learning behavior.

## 3. Results

This section presents the experimental results obtained with the proposed word-level contactless typing framework. The experiments were designed to evaluate whether words can be reliably distinguished using only finger motion patterns, without character-level decoding or explicit key localization. We first analyze performance on a physical keyboard surface under controlled conditions (Section 3.1), and then investigate generalization to non-keyboard planar surfaces (Section 3.2).

### 3.1. Word Identification on a Physical QWERTY Keyboard

All experiments reported in this section use word sets derived from the MacKenzie–Soukoreff phrase set [25], which is widely adopted as a benchmark corpus in text entry and typing performance research. Individual words were extracted from the phrase corpus after removing punctuation and eliminating duplicates. To reduce lexical bias and avoid over-representation of specific initial letters or word patterns, the resulting word pool was constructed to achieve an approximately uniform distribution over the alphabet. The selected words vary naturally in length, phonetic structure, and letter composition, reflecting realistic vocabulary encountered in everyday text entry tasks.

From this pool, subsets of different sizes (25, 50, 100, and 200 words) were sampled depending on the experimental condition. The largest evaluation set consists of 200 distinct words and is used to assess scalability and discriminability under a substantially increased vocabulary size. The complete list of the 200 words used in the full-scale evaluation is provided in Table 2, enabling reproducibility and transparent assessment of lexical diversity.

**Figure 6:**
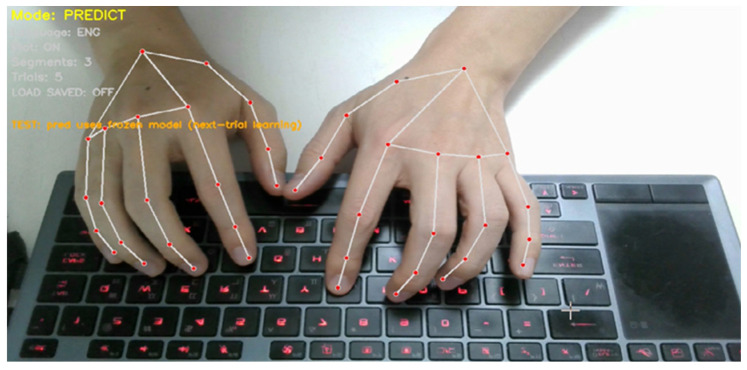
Example of word-level contactless typing over a physical QWERTY keyboard. Hand and finger landmarks are tracked in real time using a single RGB camera while the user performs natural typing motions. The system captures spatiotemporal finger trajectories without relying on key localization or character-level decoding.

Figure 6 illustrates the experimental condition used in this section. During evaluation, users performed natural typing motions over a physical QWERTY keyboard while a single RGB camera mounted above the keyboard captured hand and finger movements. Hand landmarks were tracked in real time and converted into word-level motion representations, without performing explicit key localization or character-level recognition. This setup ensures that all results reported in this section reflect discrimination based solely on finger motion patterns observed during realistic typing behavior.

Unless otherwise stated, recognition performance is reported as Top-1 accuracy, defined as the fraction of correctly predicted words among all tested trials. Typing speed is reported in words per minute (WPM), computed from the elapsed time between motion onset and termination for each word entry.

Overall, this section evaluates whether word-level contactless typing motions captured by a single RGB camera can reliably discriminate words when users perform natural QWERTY typing actions on a physical keyboard. Unless otherwise stated, the system employs the proposed direction-aware motion representation with online multi-prototype learning, and all recognition results are computed at the word level.

#### 3.1.1. Effect of Training Repetitions

We first examined how recognition accuracy changes as the number of training repetitions increases under a fixed temporal segmentation setting. In this experiment, the number of temporal segments was fixed to 3, the vocabulary size was fixed to 200 words, and typing was performed at a “normal” speed of approximately 22–25 WPM. After each training repetition, recognition accuracy was measured on the same 200-word set under the trial protocol.

Overall, recognition accuracy improved steadily as training repetitions increased. This trend indicates that repeated exposure to the same word motions leads to more consistent recognition performance under the proposed protocol. Notably, the improvement became more pronounced after the third repetition, suggesting that multiple exposures are beneficial for capturing natural execution variability at scale.

**Figure 7:**
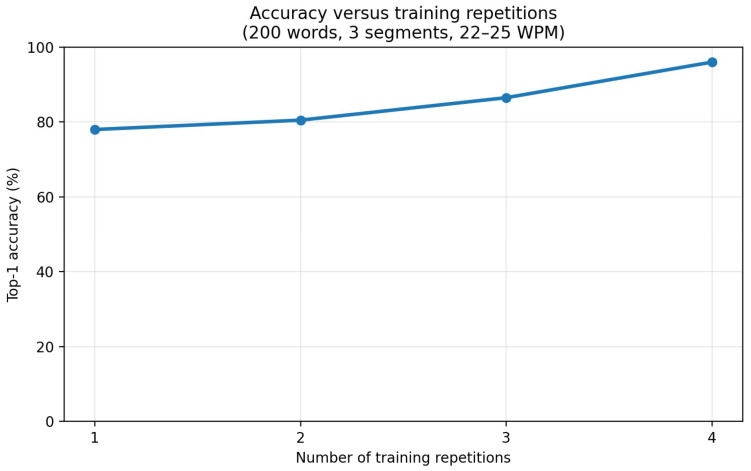
Accuracy versus training repetitions under 3-segment temporal encoding (200-word set, 22–25 WPM).

Figure 7 illustrates the recognition accuracy as a function of the number of learning passes for the 200-word vocabulary condition. Accuracy increased from 78.0% after the first pass to 80.5% after the second pass and 86.5% after the third pass, reaching 96.0% after the fourth pass. These results show a clear improvement in recognition accuracy across repetitions in a repeated closed-vocabulary setting. The observed gains may reflect a combination of prototype refinement and increased consistency in user execution across trials. All recognition results reported in this section, including the peak accuracy of 96.0%, were obtained from repeated trials conducted by a single user under a fixed experimental configuration.

#### 3.1.2. Sensitivity to Temporal Segmentation (Number of Segments)

We next analyzed how sensitive word recognition performance is to the number of temporal segments used in the proposed feature construction. In this experiment, the vocabulary size was fixed to 200 words, typing speed was maintained at 22–25 WPM, and the model was trained with one repetition. We varied the number of temporal segments from 1 to 5 and evaluated recognition accuracy for each setting.

**Figure 8:**
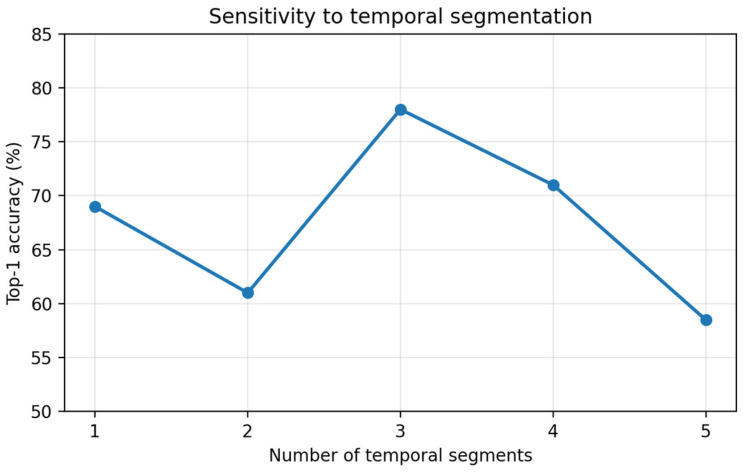
Accuracy as a function of the number of temporal segments (1–5) under the fixed protocol (200-word set, 22–25 WPM, 1 training repetition).

Figure 8 and Table 3 summarizes the results. Accuracy showed a clear dependence on segmentation granularity, reaching its maximum at 3 segments (78%). When the motion was encoded too coarsely (1 segment: 69%), performance decreased, suggesting that a single aggregated temporal representation tends to collapse distinct phases of typing-like motion into a less discriminative descriptor. Conversely, accuracy also dropped when segmentation was overly fine (5 segments: 58.5%), indicating that excessive partitioning amplifies timing inconsistency and increases sensitivity to small temporal misalignments across repetitions. Intermediate settings (2 segments: 61%, 4 segments: 71%) did not outperform the 3-segment configuration, reinforcing that an appropriate balance between temporal resolution and robustness is required for stable word-level prototype matching.

#### 3.1.3. Sensitivity to Typing Speed

To test robustness to execution speed, we compared recognition accuracy under three typing-speed regimes: slow (15–18 WPM), normal (20–23 WPM), and fast (27–28 WPM). The vocabulary size and evaluation protocol were kept consistent across conditions, and the model was trained and evaluated separately under each speed setting.

Recognition accuracy peaked at the normal speed range (86%) and decreased for both slower (58%) and faster (66%) typing. This trend suggests that the learned prototypes and the underlying motion feature representation are most stable when finger movements follow a natural typing rhythm. Very slow motions may include pauses and micro-adjustments that introduce additional variability, whereas very fast motions may compress temporal structure and reduce segmentation consistency.

**Figure 9:**
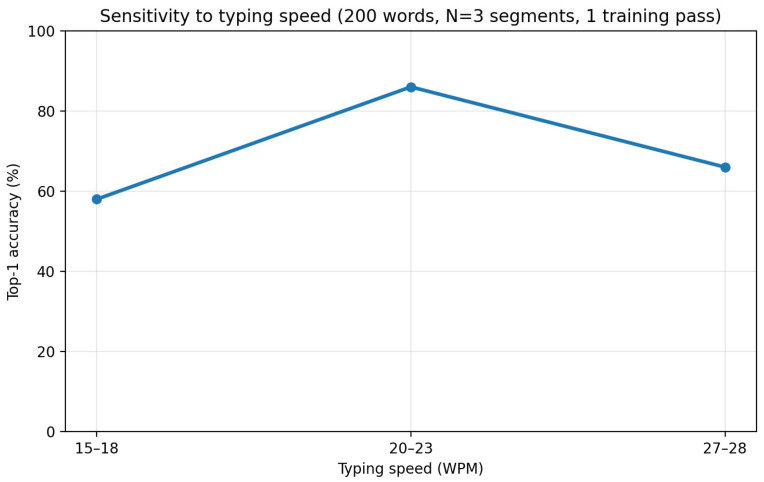
Accuracy across typing speed regimes: slow (15–18 WPM), normal (20–23 WPM), and fast (27–28 WPM).

Figure 9 illustrates the relationship between typing speed and recognition accuracy, revealing a clear performance peak at normal typing speeds and symmetric degradation for slower and faster executions.

#### 3.1.4. Sensitivity to Vocabulary Size (Number of Words)

We finally investigated how recognition performance scales as the vocabulary size increases. Using the best-performing temporal configuration identified in the previous analysis (3 segments) and a normal typing speed of 22–25 WPM, the system was trained once and evaluated on vocabularies consisting of 25, 50, 100, and 200 words.

**Figure 10:**
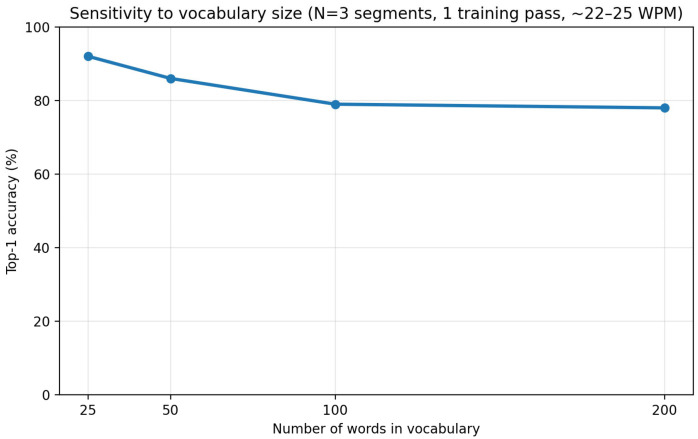
Recognition accuracy as a function of vocabulary size (25–200 words) under 3-segment temporal encoding, normal typing speed (22–25 WPM), and one training repetition.

Figure 10 illustrates the relationship between vocabulary size and recognition accuracy. A clear monotonic decrease in accuracy is observed as the number of words increases. When the vocabulary was limited to 25 words, the system achieved a high accuracy of 92%, indicating that word-level motion patterns can be reliably discriminated when class competition is minimal. As the vocabulary expanded to 50 and 100 words, accuracy gradually declined to 86% and 79%, respectively. This trend reflects the increasing overlap among motion patterns as more words with partially similar finger trajectories are introduced.

Notably, performance saturated near 78% at the 200-word scale, suggesting that the degradation is not linear with vocabulary size. Instead, the system appears to reach a stable operating regime in which additional words introduce diminishing marginal confusion. This behavior implies that a substantial portion of motion ambiguity arises once the vocabulary exceeds a moderate size, beyond which the learned multi-prototype representations maintain a consistent level of discrimination.

### 3.2. Word Identification on a Flat Surface (No Physical Keyboard)

This subsection evaluates the generalization performance of the proposed framework when users perform typing-like finger motions without a physical keyboard, i.e., over a flat desk surface. To ensure that stable word-level motion prototypes were available prior to flat-surface testing, the system was first trained on the same 50-word vocabulary through five repetitions on a physical keyboard. After this training phase, the prototype sets were frozen, and all flat-surface evaluations were performed in prediction-only mode without any further model updates. This design isolates cross-surface generalization and prevents confounding effects from additional learning in conditions where physical key contact is absent.

**Figure 11:**
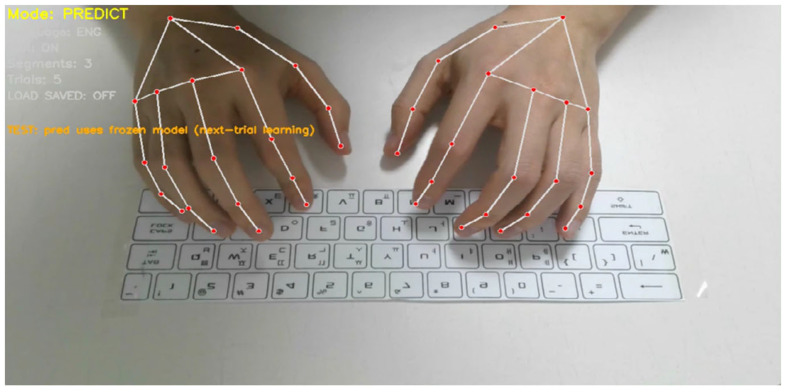
Flat-surface typing setup with a keyboard-shaped visual guide. A printed QWERTY layout provides spatial reference while removing physical key travel and tactile feedback.

**Figure 12:**
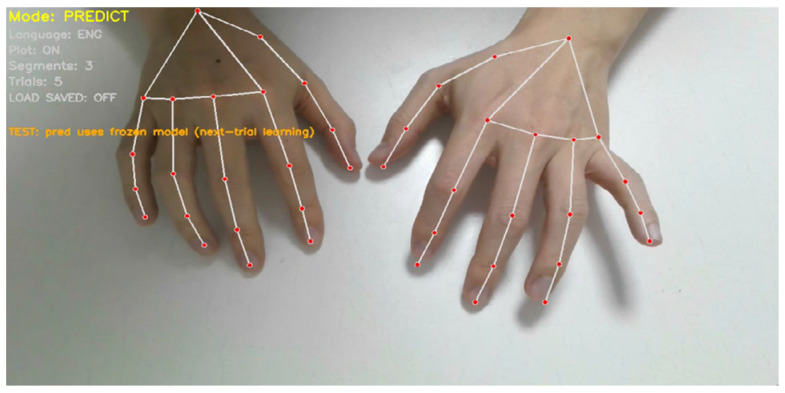
Flat-surface typing setup with no visual guide. Typing-like finger motions are performed on a blank surface, with only the hands visible in the camera view.

Two flat-surface conditions were considered, differing only in the availability of visual spatial guidance. In the first condition, a keyboard-shaped paper matching the size and layout of a standard QWERTY keyboard was placed on the desk to provide a visual reference, while physical key travel and tactile feedback were removed (Figure 11). In the second condition, typing-like motions were performed on a blank flat surface with no keyboard outline or visual cue, leaving only the hands visible in the camera view (Figure 12). By comparing these two conditions, the experiment isolates the effect of explicit visual layout cues from physical key contact and evaluates whether motion patterns learned on a physical keyboard transfer to increasingly unconstrained surface settings.

Because the model was not adapted online during flat-surface testing, changes in recognition accuracy across repetitions primarily reflect user-side familiarization and skill acquisition in producing consistent typing-like motions without tactile reference, rather than within-session prototype refinement. Table 4 summarizes the word identification accuracy as a function of evaluation repetition count under the frozen-model protocol.

As shown in Table 4, the keyboard-shaped visual guide yields consistently higher accuracy, particularly during early repetitions. With visual layout guidance (Figure 11), accuracy improves from 78% to 86%, suggesting that approximate spatial cues help the user reproduce finger trajectories that remain compatible with prototypes learned on a physical keyboard, even without key travel or tactile confirmation.

In contrast, the no-guide condition (Figure 12) shows substantially lower initial accuracy (62%), reflecting the challenge of initiating stable typing-like motions without any external spatial reference. However, accuracy increases rapidly across repetitions, reaching 80% by the fourth repetition. Under the frozen-model protocol, this improvement is best interpreted as a practice effect: the user progressively learns to generate more repeatable spatiotemporal finger motions and implicit spatial alignment in the absence of tactile and visual guidance, thereby increasing consistency with the pre-learned prototypes.

Importantly, because both conditions used the same frozen prototype set learned from identical prior keyboard training, the observed performance differences can be attributed primarily to the availability of visual guidance and the degree of surface constraint, rather than insufficient word learning. The narrowing gap across repetitions further suggests that intrinsic word-specific motion patterns can be reproduced with increasing consistency even when explicit spatial cues are removed, supporting the robustness of the proposed word-level representation under progressively unconstrained interaction settings.

### 3.3. Statistical Considerations and Evaluation Scope

The results presented in this section are primarily intended to examine system-level feasibility, learning behavior, and generalization trends of the proposed word-level contactless typing framework, rather than to establish population-level statistical significance.

The current evaluation focuses on repeated word-entry trials performed by a single user under controlled conditions, with performance reported as trial-wise word recognition accuracy and typing speed (WPM). Because each condition yields a single accuracy value per repetition, conventional inferential statistical analyses (e.g., hypothesis testing with *p*-values) are not applicable in a strict sense. Instead, the results are interpreted in terms of consistent performance trends across repetitions, vocabulary sizes, and surface conditions.

This evaluation strategy is intentionally aligned with the goals of the proposed framework. The central question addressed in this paper is not whether one interaction technique statistically outperforms another across a population, but whether word-level motion patterns can be learned, recalled, and incrementally refined using only a single RGB camera, without character-level decoding or key localization. Accordingly, the reported trends—such as accuracy improvement with repeated exposure, sensitivity to temporal segmentation, performance variation across typing speeds, vocabulary scaling behavior, and flat-surface transfer—demonstrate internal consistency and stable performance behavior of the proposed approach within the evaluated single-user, fixed experimental configuration.

In addition, the use of a trial-delayed learning protocol ensures that recognition accuracy measured within each trial reflects genuine generalization to previously unseen executions, rather than immediate memorization effects. As a result, improvements observed across trials can be attributed to accumulated learning rather than within-trial adaptation, providing a conservative estimate of system performance.

For future studies, the proposed framework naturally lends itself to broader statistical evaluation. Extensions may include multi-participant experiments, repeated sessions across days, and cross-user generalization analyses. In such settings, recognition accuracy may be reported as mean ± standard deviation or confidence intervals, and appropriate statistical tests—such as repeated-measures ANOVA or non-parametric alternatives (e.g., the Friedman test)—can be applied to compare surface conditions, segmentation settings, or learning strategies. Effect size measures (e.g., Cohen’s *d*) would further help quantify practical differences between conditions.

Nevertheless, the present results establish a critical foundation: word-level contactless typing based solely on monocular vision is feasible and learnable under the constrained execution variability observed within the evaluated single-user, fixed-camera setup. These findings justify more extensive statistical validation in future work and motivate discussion of the broader implications and limitations of the proposed approach, which are addressed in the following section.

## 4. Discussion

This study demonstrates that natural QWERTY typing motions can be directly recognized at the word level using only a single RGB camera, without performing character recognition or explicit key localization. By reframing contactless text entry as a word-level motion recall problem, the proposed approach departs from the dominant key-centric and character-centric paradigms in prior vision-based typing research.

Across the experimental results, a consistent trend emerges that repeated execution leads to progressive performance improvement under a range of tested input conditions. Within the tested fixed-camera configuration, this behavior suggests that the proposed representation captures relatively stable spatiotemporal characteristics of finger motion under the limited execution variability present in the current evaluation. In particular, the flat-surface transfer experiments reveal that even when both tactile feedback and explicit visual layout cues are removed, word-level motion patterns can still be recalled and refined through repeated exposure. This suggests that the proposed framework relies primarily on relative finger motion structure, rather than absolute key positions. Although the proposed representation preserves coarse temporal ordering through uniform segmentation of each word-level motion sequence, it intentionally summarizes finger motion within each segment using direction-aware displacement accumulation. As a result, fine-grained temporal structure within individual segments is not explicitly encoded, and distinct trajectories may partially collapse into similar representations. This design reflects a deliberate trade-off between representational compactness and temporal detail, and was found sufficient for feasibility evaluation of word-level motion recall under repeated exposure. Accordingly, the sensitivity analysis in this study focuses primarily on the segmentation parameter N, as it directly governs the temporal resolution and stability of the proposed word-level motion representation. Other parameters, including prototype adaptation rates, distance thresholds, and termination-related settings, were intentionally fixed to isolate the effect of segmentation granularity during feasibility evaluation. A more comprehensive sensitivity analysis covering these parameters remains an important direction for future work.

Such behavior is especially relevant in monocular vision settings, where limited spatial resolution, occlusion, and viewpoint variation make precise key-level discrimination inherently fragile. Whereas small localization errors in character-based systems can immediately propagate into word-level failures, the holistic nature of word-level motion patterns allows recognition to remain stable if the overall trajectory structure is preserved. From this perspective, the proposed formulation can be interpreted as a potential structural mitigation of some practical limitations observed in vision-only contactless input under constrained sensing conditions. The online multi-prototype learning strategy plays a critical role in accommodating natural execution variability. Instead of forcing all repetitions of a word into a single averaged template, the system incrementally builds a set of representative motion prototypes, adding new ones only when novel patterns are observed. This design enables the model to absorb intra-word variability while avoiding overfitting to transient fluctuations. The use of a trial-delayed learning protocol further helps reduce immediate memorization effects within a trial and supports more consistent evaluation across repeated executions, rather than immediate memorization within a trial. While the multi-prototype mechanism enables adaptation by allowing memory growth, this study does not explicitly disentangle performance gains due to user practice from those due to model growth. Quantitative analysis of prototype counts and their direct relationship to recognition accuracy is therefore left for future work.

The flat-surface experiments also provide insight into the role of visual guidance. While the presence of a keyboard-shaped visual guide improves initial performance, the performance gap between guided and unguided conditions decreases substantially with repeated execution. This finding suggests that internalized motor memory associated with QWERTY typing remains sufficiently consistent even when external spatial references are removed, and that word-level motion representations can adapt to reduced external spatial cues within the tested experimental setup.

Despite these encouraging results, several limitations should be acknowledged. The current evaluation is based on a single user and a limited number of sessions, and thus does not support population-level statistical generalization. In addition, the proposed framework assumes a closed vocabulary and does not directly address character-level editing or open-vocabulary input. These limitations, however, reflect deliberate design choices aimed at isolating and validating the core feasibility of word-level motion recognition under minimal sensing assumptions, rather than shortcomings of the approach itself. Accordingly, the present results should be interpreted as a feasibility validation rather than evidence of general deployability. The absence of direct baseline comparisons in this study reflects a deliberate design choice rather than an oversight. The primary objective is not to benchmark recognition accuracy against character-level or sequence-alignment-based pipelines (e.g., DTW or HMM with language-model support), but to examine whether word-level motion recall is feasible under minimal monocular sensing assumptions.

From an experimental perspective, the current study relies on fixed indoor lighting and a relatively controlled background environment. While robust hand tracking was achieved under these conditions, evaluating the system under varying illumination, shadows, or increased background clutter remains an important direction for future work.

In this paper, scalability is not intended to be interpreted in the deployment sense. Rather, it refers to the ability of the proposed system to maintain internally consistent recognition behavior as vocabulary size and associated memory increase within the constrained single-user, fixed-camera experimental regime evaluated in this study.

From a scalability standpoint, word-level matching in the current implementation scales linearly with the number of stored prototypes, which would introduce increasing computational overhead as the vocabulary grows. Addressing this limitation would require additional strategies such as candidate pruning, hierarchical matching, or approximate nearest-neighbor search, which are left as directions for future work.

## 5. Conclusions

This paper presented a novel contactless text entry framework that recognizes word-level typing motions directly from monocular RGB video, without relying on character recognition, key localization, or specialized hardware. By modeling each word as a distinctive spatiotemporal finger motion pattern and learning these patterns through online multi-prototype adaptation, the proposed system enables reliable word identification within the tested configuration, even under substantial spatial uncertainty.

Experimental results demonstrate that the framework can progressively learn and recall word-level motion patterns across repeated executions and can transfer learned representations to flat-surface conditions where tactile and visual layout cues are limited or absent. These findings demonstrate the feasibility of reframing contactless typing as a word-level motion recall problem and suggest that such a formulation offers structural advantages over conventional character-centric approaches in vision-based settings.

More broadly, this work introduces an alternative component-level design perspective for vision-only input, in which word-level motion recall can complement conventional character-centric techniques under constrained sensing conditions. Rather than serving as a standalone text-entry solution, the proposed approach is best interpreted as a building block for restricted command vocabularies, predictive phrase input, or calibration-assisted interaction scenarios.

## Figures and Tables

**Table 1:** System parameters used in the proposed method.

Parameter	Description	Value
α	Landmark smoothing factor	0.8
$\tau_{start}$	Motion start threshold (px)	2
$\tau_{jit}$	Jitter suppression threshold (px)	2
N	Number of temporal segments	1, 2, 3, 4, 5
Feature dimension	Motion feature dimensionality	32×N
(η)	Prototype update gain	0.5
$\tau_{new}$	New prototype distance threshold	35
Distance metric	Prototype matching	Euclidean

**Table 2:** Word set used in the 200-word evaluation condition.

acceptance acutely airport amount anniversary aspirations assault athletes aware baloney bathroom beautiful birthday blazing bloodshed blocked boring breaks camera camping carpet chamber chemical chlorine coalition collapse complicated cookies daring decisions deed delivery destruction diction disgusting driveways drugs edition electrical encounter encouragement environment equation etiquette exercise expenses explosion facts fast field fingers flashing forest fuel fueling force gallery gas generation glance glasses golfers governments guidelines hair hand hard historic house hours human imagination immediately important independent indication investigate interesting island jacket jacketed joke join just justice journey judgment keeps key kindness kitchen knocking knowledge known lagoon largest laughter leather light listen looks losers machinery mask maximum medieval meeting midnight mortgage nation never nobody noon nothing number objectives obligations observation oceans occasional objective opera order painting patio penalty phenomenon picture presence priorities proposal punishment quality quantity quarter question queue quick quite race rain receipts registration relations response Roman round saddles santa seasoned security shouting spectacular stability subdivisions summit taxation tennis thousand tickets traffic traditional transaction treasury turn universally universities understood until use usually utility valid variety very victims voice voters volume window winter winners wore words work wrong yard yap year yesterday young your youth zero zest zigzag zinc zodiac zone zoom

**Table 3:** Segmentation sensitivity results (200-word set, 22–25 WPM).

Segments (S)	Accuracy (%)	Avg WPM
1	69.0	26.81
2	61.0	26.04
3	78.0	26.83
4	71.0	27.36
5	58.5	24.99

**Table 4:** Word identification accuracy (%) on flat-surface typing after five prior training repetitions on 50-word vocabulary.

Evaluation Repetition	Keyboard-Shaped Visual Guide	No Visual Guide
1	78.0	62.0
2	80.0	68.0
3	82.0	78.0
4	86.0	80.0

## Data Availability

The original contributions presented in the study are included in the article, further inquiries can be directed to the corresponding author.

## Abbreviations

The following abbreviations are used in this manuscript:

WPM	Words Per Minute
CNN	Convolutional Neural Network
RNN	Recurrent Neural Network
HCI	Human–Computer Interaction
QWERTY	Standard keyboard layout derived from the first six letters
MCP	Metacarpophalangeal Joint
PIP	Proximal Interphalangeal Joint
DIP	Distal Interphalangeal Joint
TIP	Fingertip Joint
